# Radiotherapeutic Outcomes for Localized Primary Rectal Mucosa-Associated Lymphoid Tissue Lymphoma: A Consecutive Case Series of Three Patients

**DOI:** 10.7759/cureus.22307

**Published:** 2022-02-16

**Authors:** Atsuto Katano, Kenta Takeuchi, Hideomi Yamashita

**Affiliations:** 1 Radiology, The University of Tokyo Hospital, Tokyo, JPN

**Keywords:** non-hodgkin's lymphoma, mucosa-associated lymphoid tissue (malt), conventional radiotherapy, gi oncology, radiotherapy (rt)

## Abstract

Gastrointestinal malignant lymphoma is uncommon and accounts for a small proportion of all gastrointestinal neoplasms. Primary rectal extranodal marginal zone lymphoma of mucosa-associated lymphoid tissue (MALToma) is a rare type of intestinal lymphoma. Here, we report about three patients (two females, one male) with localized rectal MALToma who were treated with external beam radiation therapy (EBRT). The median age of the patients was 59 years (range: 50-67 years). Chemotherapy or eradication therapy was not performed before EBRT. All patients received a radiation dose of 30 Gy in 15 fractions using X-ray photon beams. Pathological examination confirmed complete remission of rectal MALToma after EBRT in all patients. At approximately five years after EBRT, none of the patients showed any evidence of recurrence of rectal MALToma. The use of EBRT resulted in excellent disease control, and no severe radiation-induced toxicity was observed. These results suggest that EBRT is a useful treatment modality for primary rectal MALToma.

## Introduction

Malignant lymphoma of the gastrointestinal tract is rare, accounting for less than 5% of all gastrointestinal neoplasms [1]. The most common site of primary malignant lymphoma of the gastrointestinal tract is the stomach (60%), followed by the small intestine, large intestine, and esophagus [2]. The predominant subtype of gastrointestinal lymphomas is the B-cell type, and the most common histologic types are extranodal marginal zone lymphoma of mucosa-associated lymphoid tissue (MALToma) and diffuse large B-cell lymphoma [3]. Their etiology remains unknown, and potential risk factors include celiac disease, bacterial infection, viral infection, immunosuppressive agents, and inflammatory bowel disease [4]. The clinical presentation of gastrointestinal lymphoma is non-specific with symptoms such as abdominal discomfort, loss of appetite, hemorrhage, and diarrhea [5].

Primary rectal MALToma is an extremely rare disease with no consensus on the treatment strategy. There is insufficient reliable evidence to select an appropriate treatment. Here, we report patients with early-stage primary rectal MALToma who were treated with external beam radiation therapy (EBRT).

## Case presentation

A total of three patients who were treated for rectal MALToma between December 2014 and November 2019 are included in this case series. The diagnosis was histologically confirmed using colonoscopic biopsy of the rectum, and the clinical staging was classified according to the Lugano classification [6]. Localization of the disease was confirmed using upper gastrointestinal endoscopy and positron emission tomography, and bone marrow puncture confirmed the absence of distant metastases and infiltration of the bone marrow. We only included the initial treatment cases and excluded patients with residual disease after prior treatment or those who had a recurrence. None of the patients had any history of chemotherapy or eradication therapy before EBRT. Treatment-related adverse events were retrospectively graded according to Common Terminology Criteria for Adverse Events.

Computed tomography (CT) was performed with a 5-mm slice for EBRT planning, and the CT images were transferred to the treatment planning system. The clinical target volume (CTV) included the evident tumor on the planning CT image and mesorectal lymph nodes. The planning target volume was defined as 5-mm uniform expansion around CTV. The total radiation dose for all patients was 30 Gy in 15 fractions administered five days per week.

There were two females and one male with a median age of 59 years (range: 50-67 years). The clinical details of the three patients are displayed in Table 1 with a median follow-up of 57.5 months (range: 56.7-59.7 months). None of the patients had systemic symptoms at presentation, including fever, weight loss, and night sweats. Serum lactate dehydrogenase levels were not elevated in two patients (Case A and B), and these patients were classified as the low-risk group, as assessed by the MALT-Lymphoma International Prognostic Index [7]. The *Helicobacter pylori* test results were negative in all patients. Colonoscopy and biopsy were performed for post-radiotherapy evaluation, and all patients were in complete remission two to four months after radiotherapy. All three patients underwent annual colonoscopy and biopsy surveillance, and no relapse occurred during the observation period.

**Table 1:** Patient characteristics and treatment outcomes. KPS: Karnofsky Performance Status; LDH: lactate dehydrogenase; AV: anal verge; NA: not assessed

Case	Age (years)	Sex	KPS	Lugano classification	LDH (U/L)	Location	Follow-up period	Status of last follow-up
A	67	Female	100	I (diameter 1 cm)	396	Ra-Rb (AV 12 cm)	59.7 months	Relapse-free survival
B	59	Female	100	I (diameter 3 cm)	188	Rb (AV 2 cm)	57.5 months	Relapse-free survival
C	50	Male	90	I (diameter 2 cm)	NA	Rb (AV 3 cm)	56.7 months	Relapse-free survival

Case A

**Figure 1:**
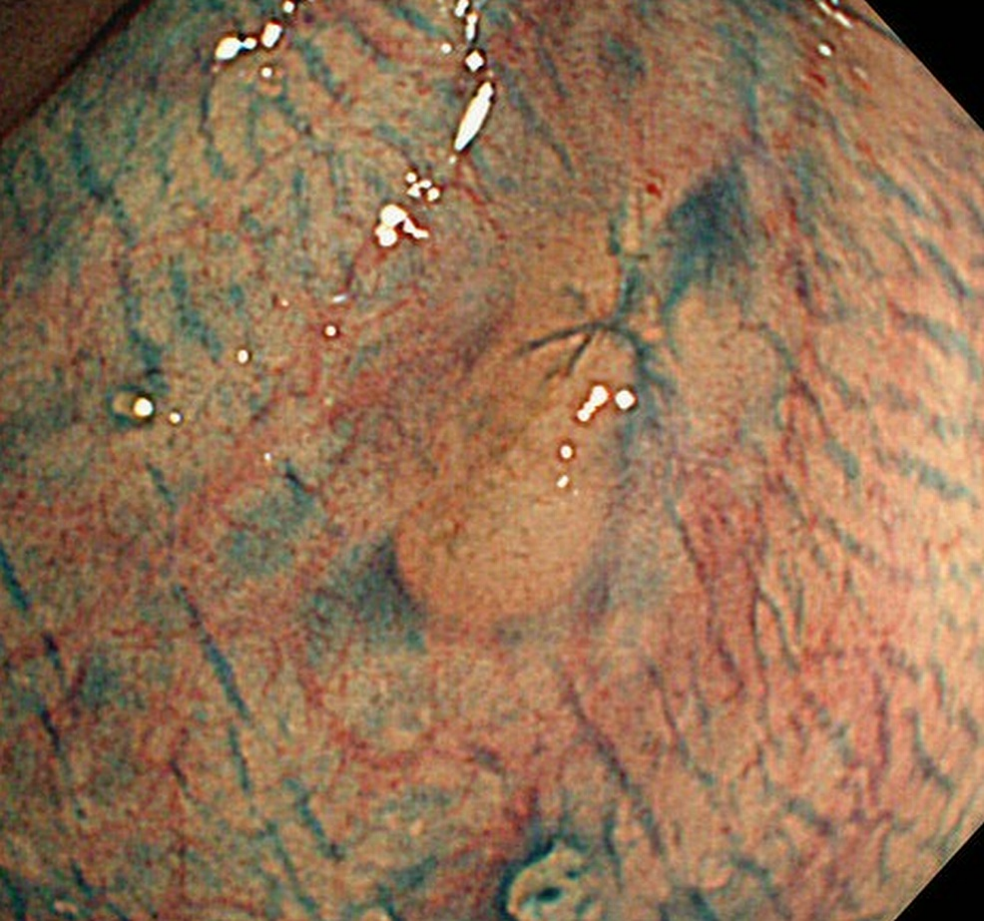
Colonoscopic image. Colonoscopy shows a submucosal tumor of approximately 1 cm in diameter in the rectum.

A 67-year-old female was referred to our hospital because an occult blood test was positive during an annual routine medical check-up. She had a medical history of broncho-pneumonia 27 years ago. She had a habit of drinking wine a couple of times per week but never smoked. Colonoscopy revealed a submucosal tumor (SMT) of approximately 1 cm in diameter located 12 cm from the anal verge (Figure 1). A biopsy was performed, which identified MALToma. EBRT was administered for three weeks, and she experienced diarrhea (grade 1) and leukopenia (grade 1), but no antidiarrheal drug was needed. Four months after EBRT, the SMT disappeared, and the biopsy showed remission of MALToma.

Case B

A 59-year-old female complained of rectum hemorrhage for several months before presenting to our hospital. She had been suffering from chronic constipation and abdominal discomfort for a long time. She did not drink or smoke. Colonoscopy disclosed a bump that was 3 cm in diameter and covered with intact mucosa in the rectum. During EBRT, she experienced diarrhea (grade 2) and fatigue (grade 1) without any hematological adverse event. After EBRT, these adverse events were improved gradually and remission of MALToma was achieved.

Case C

**Figure 2:**
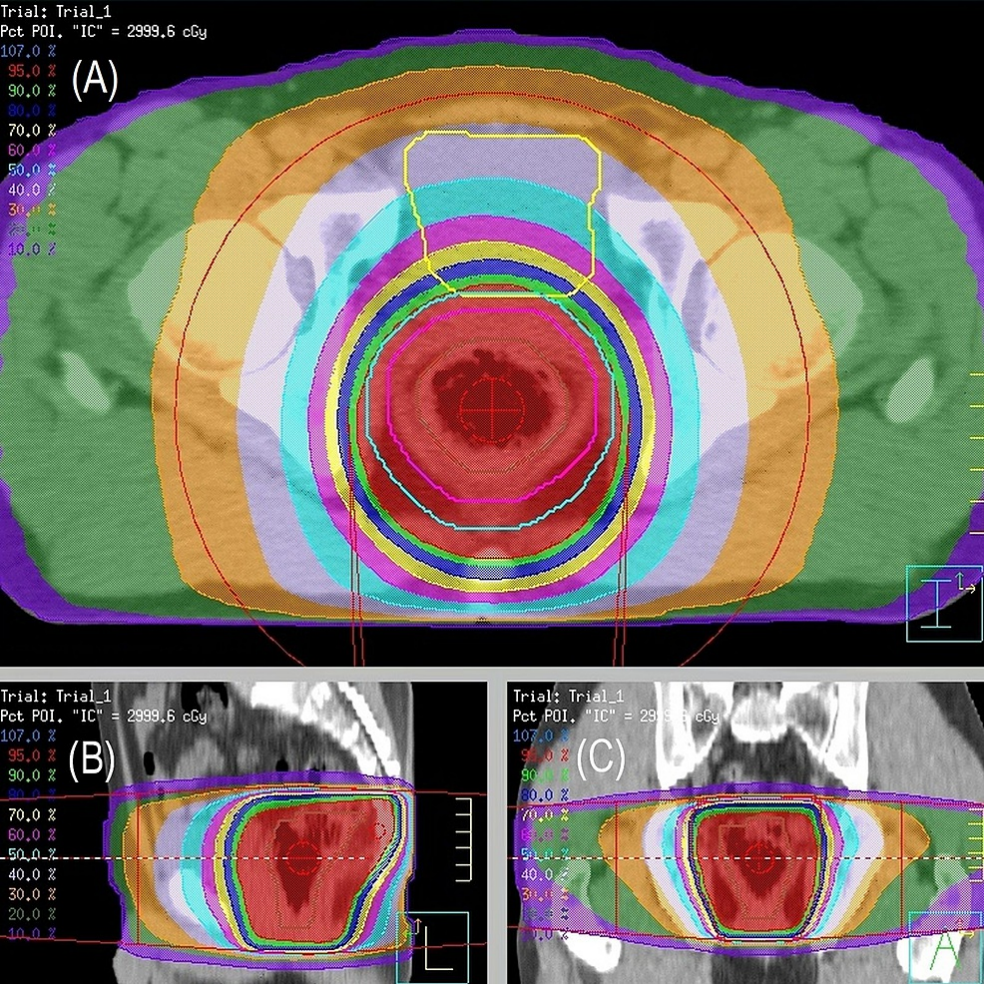
Radiation treatment planning. The light blue line indicates planning target volume, and the red region indicates the 95% isodose area of the prescribed dose (30 Gy). (A) Axial Plane, (B) sagittal plane, and (C) coronal plane.

A 50-year-old male was pointed out fecal occult blood during an annual medical check-up. He had no medical history of systemic disease. During the clinical examination for the occult bleeding, a 3 cm SMT in the lower rectum was noted on endoscopic examination. He underwent endoscopic biopsy, and the final diagnosis was MALToma. After confirming no distant metastasis, EBRT was delivered at a total dose of 30 Gy in 15 fractions (Figure 2). There were no treatment-related adverse events, excluding slight diarrhea (grade 1) during EBRT. After EBRT, remission of MALToma was obtained, and there was no sign of recurrence for over four years.

## Discussion

While the gut is responsible for the digestion and absorption of nutrients, it also plays an important role in maintaining immune homeostasis [8]. Gut-associated lymphoid tissue (GALT) is known as the largest immunologic organ in the body and considerably contributes to the immune system of the whole body [9]. Intestinal MALToma is considered to develop from GALT, and the stomach is the most common site of intestinal lymphoma [10]. Excellent clinical outcomes have been achieved using EBRT in patients with gastric MALToma [11]. Watanabe et al. compared the treatment outcomes of radiotherapy for non-gastric intestinal MALToma with those of gastric MALToma [12]. There was no significant difference in overall survival and disease-free survival between gastric and non-gastric MALToma.

Won et al. reported that the most frequently used therapeutic modalities for colorectal MALToma are surgery and endoscopic resection [13]. They reported that both treatment strategies achieved a complete remission rate of over 90%. Lymph node dissection should be considered in cases in which radical surgical resection is performed because regional lymph node involvement has been reported in many cases [14]. Endoscopic surgery is a less invasive treatment procedure than conventional surgery and is an organ-sparing resection technique for early-stage malignant disease. Han et al. reported a successful case of endoscopic resection of submucosal protrusion of rectal MALToma using endoscopic submucosal dissection [15].

*Helicobacter pylori *eradication therapy is widely known as a safe and effective treatment for gastric MALToma. Moreover, Defrancesco et al. pointed out that eradication therapy can be effective in non-gastric MALToma cases [16]. Ito et al. reported a case of primary rectal MALToma regression one month after eradication therapy comprising lansoprazole, amoxicillin, and clarithromycin [17]. Kelly et al. reviewed the English literature and reported that the positive rate for *Helicobacter pylori *test in primary rectal MALToma patients is 43% [18]. According to their review, although the primary rectal MALToma temporally responds well through eradication therapy, as many as 37% of patients required second-line treatment during long-term follow-up.

## Conclusions

There is no current consensus regarding the treatment of primary rectal MALToma. In this study, we found that EBRT resulted in a good response to treatment and that the lesions were controlled for approximately five years in all patients without severe adverse events. Our results suggest that EBRT is a useful treatment option for primary rectal MALToma.
